# The co.LAB Generic Framework for Collaborative Design of Serious Games: Development Study

**DOI:** 10.2196/28674

**Published:** 2021-07-02

**Authors:** Dominique Jaccard, Laurent Suppan, Eric Sanchez, Audrey Huguenin, Maxence Laurent

**Affiliations:** 1 Media Engineering Institute University of Applied Sciences Western Switzerland Yverdon Switzerland; 2 Division of Emergency Medicine, Department of Anesthesiology, Clinical Pharmacology, Intensive Care and Emergency Medicine University of Geneva Hospitals and Faculty of Medicine Geneva Switzerland; 3 Technologies de Formation et Apprentissage Educational Technologies, Faculty of Psychology and Educational Sciences University of Geneva Geneva Switzerland

**Keywords:** serious game, educational game, simulation game, design, design framework, methodology, collaborative design, collaborative web platform

## Abstract

**Background:**

Serious games are increasingly used at all levels of education. However, research shows that serious games do not always fulfill all the targeted pedagogical objectives. Designing efficient and engaging serious games is a difficult and multidisciplinary process that requires a collaborative approach. Many design frameworks have been described, most of which are dedicated to the development of specific types of serious games and take the collaborative dimension into account only to a limited extent.

**Objective:**

Our aim was to create a generic serious game design framework that could be adapted to all kinds of serious games and implemented in a collaborative web platform.

**Methods:**

We combined the results of a literature review with our experience in serious game design and development to determine the basic building blocks of a collaborative design framework. We then organized these building blocks into categories and determined the features that a generic design framework should include. Finally, based on the paradigm of complex systems and systemic modelling, we created the co.LAB generic design framework and specifications to allow its implementation in a collaborative web platform.

**Results:**

Based on a total of 10 existing design methodologies or frameworks, 23 building blocks were identified and represent the foundation of the co.LAB framework. These blocks were organized into 5 categories: “context and objectives,” “game design,” “mechanics,” “learning design,” and “assessment.” The arrangement by categories provides a structure that can be visualized in multiple and complementary ways. The classical view links game and learning design while other views offer project, systemic, and process visualizations. For the implementation of the co.LAB framework in a web platform, we propose to convert the building blocks into “cards.” Each card would constitute a collaborative working space for the design of the corresponding block. To make the framework adaptive, cards could be added, adapted, or removed according to the kind of serious game intended. Enhancing the visualization of relationships between cards should support a systemic implementation of the framework.

**Conclusions:**

By offering a structured view of the fundamental design elements required to create serious games, the co.LAB framework can facilitate the design and development of such games by virtue of a collaborative, adaptive, and systemic approach. The different visualizations of the building blocks should allow for a shared understanding and a consistent approach throughout the design and development process. The implementation of the co.LAB framework in a collaborative web platform should now be performed and its actual usability and effectiveness tested.

## Introduction

### Background

The term “serious games” is used in many different meanings, and there is still no strict agreement on what it exactly encompasses. In broad definitions, such as Zyda’s [1] or in Michael and Chen [2], serious games may be used for achieving any kind of nonentertainment objectives, including education, health, public policy, or communication. In other definitions, such as those by Abt [3] or Loh et al [4], serious games are restricted to educational and training objectives. In this article, we use the term serious games in the sense of the definition by Loh et al [4], thus encompassing any kind of digital games or simulation created for educational or training purposes.

#### Serious Games for Educational Purposes

Serious games are effective tools to support learner-centered teaching practices [5-9], and interest in serious games has flourished at all levels of education. The COVID-19 crisis has accelerated the digitalization of education, and the use of digital educational resources such as serious games is expected to increase even further in the coming years [10]. However, research shows that serious games do not always fulfill all the targeted pedagogical objectives [11-13]. To reach its objectives, a serious game needs to successfully integrate gaming and learning aspects and be accepted by the teachers who will use it. Collaborative design of serious games is recognized as a success factor for both this integration and acceptance.

#### Collaborative Design of Serious Games

The collaborative work of a multidisciplinary team, including game developers, teachers (or trainers), and educational scientists, is required to design and develop efficient serious games [13-16]. This collaboration is recognized as a significant factor in the pedagogical relevance of the resulting development [14,17].

Integration of gaming and learning aspects, and integration of serious games into an overall pedagogical scenario, has been recognized as a key success factor [13,18,19]. Thus, the collaboration within the development team must ensure that partial contributions of different specialists who possess complementary knowledge and expertise will result in a coherent solution integrating pedagogical and playful aspects.

Collaboration in serious game multidisciplinary development teams can however be difficult [20]. Difficulties arise from the intrinsic multidisciplinarity of serious game design and from the challenging balance between game and pedagogical elements. Communication and coordination problems resulting from differences in vocabulary, background, and expectations also arise during the design and development phases [8,16,20-22]. Thus, while mandatory, collaboration in serious game design can be difficult.

#### Collaborative Web Platforms

Collaboration during serious game design could be facilitated by the use of a collaborative web platform. This platform should support typical collaborative dimensions such as mutual understanding, information pooling, communication management, group problem solving, reaching consensus, and task or time management [23,24].

For a multidisciplinary team, often geographically dispersed, a web platform may offer collaborative functionalities such as shared workspaces with a global design overview (mutual understanding), up-to-date documents (information pooling), discussion threads (communication management), voting systems (reaching consensus), and project management (task and time management). De Troyer [20] emphasized the need for this type of tool to support and stimulate the collaborative development of serious games. Regular tools usually employed for software development are not suitable for the development of serious games [20]. The main reason is that such tools were designed for software developers, not for multidisciplinary development teams incorporating noncomputer scientists [20]. Another reason is that existing platforms do not provide the necessary overview and integration needed for the development of both the serious game and its pedagogical integration [20].

Thus, collaborative web platforms may support collaborative design of serious games, but existing platforms are not suitable for that purpose.

#### Current Serious Game Design Frameworks

A collaborative platform dedicated to serious game design and development should be based on a design framework. Serious game design frameworks and methodologies are intended to provide development teams with design foundations and guidelines that support collaboration in the development of an integrated solution [8,16,25,26]. The framework implemented in a collaborative platform must allow, on the one hand, the development of the greatest number of different types of serious games and, on the other hand, be compatible and facilitate the implementation of collaborative features.

Most existing frameworks are dedicated to the design and development of specific types of educational games [16,27]. Thus, when beginning a new serious game project, the design team must choose a specific design framework and get used to it. Although not always straightforward, the task of choosing such a framework is usually rather easily feasible. However, using a framework that is too specific and not adaptable enough as the basis for a collaborative platform would force design teams to adapt to the framework, which could cause major problems. To achieve the intended serious game, the framework should be adapted to the project, rather than having to adapt the projected game to the framework.

A collaborative framework should provide the design teams with an overview of all design elements. Some existing frameworks give a broad overview of 3 or 4 categories to be considered (such as “Play, Pedagogy and Fidelity” in [8] or “Context, Pedagogy, Representation and Learner” in [28]) but do not provide a detailed view of specific design elements of each category. Few frameworks give a more detailed list of game and learning design elements, but do not offer a categorization and structured view that may enable understanding the role of the different experts and the link between elements. Most frameworks do not include the design of the pedagogical scenario into which the serious game will be implemented.

A collaborative framework should also provide a project management perspective. Project management during serious game design and development is challenging because of the difficulty of managing multidisciplinary teams and of the need to adopt an iterative process [16,29]. Project management support (task, time, resource allocation) during serious game design and development is highlighted as needed [30], but not included in existing frameworks. Most existing frameworks include some specific guidelines but do not support collaborative work nor provide practical guidance describing how the different steps of the development process should be carried out.

Thus, existing serious game design frameworks were not designed with the goal to be implemented in a web platform and present some shortcomings in that perspective. If some of the needed qualities are found in each existing framework, none of them include the complete set of necessary qualities. An ideal collaborative serious game design platform should be based on a framework that provides an overall structure with content that can be customized by the end users. It should be an adaptive framework rather than “one framework to rule them all” and thus be considered more as a general methodology (a set of tools and guidelines) rather than as a traditional framework. It should support a collaborative and interprofessional approach, as well as the possibility to view the development process from different angles, offering both a broad overview of design categories and a detailed view of design elements.

#### The co.LAB Project

The co.LAB project, which is funded by the Swiss National Science Foundation, aims at improving efficiency and relevance in serious game design and development by supporting the collaboration between all members of the multidisciplinary development team. This goal should be achieved by developing a methodological framework associated with a collaborative web platform dedicated to the co-design, co-development, and co-evaluation of serious games.

### Objectives

Our main objective was to create a methodological framework suitable for implementation into a collaborative web platform. This framework should enable the design and development of all kinds of serious games. Our secondary objective was to define guidelines and basic collaborative functionalities for the implementation of such a framework in a collaborative web platform.

## Methods

To identify the elements of a generic serious game design framework, we combined the results of a literature review with the authors’ experience in serious game development.

The literature review was based on a search in Google Scholar using the terms “serious game design” and “educational game design.” We added articles that were known by the authors and did snowballing searches from references and citations of identified articles. As the aim of the literature review was to identify the essential building blocks needed to develop the basic structure of a generic framework, we considered a systematic review unnecessary; it might have yielded more results but probably not led to the inclusion of more building blocks. We then selected the most relevant frameworks based on their suitability for our purpose. We considered a framework to be relevant when it had actually been used for the development of at least one serious game and had been described in enough detail to allow replication. While citation numbers were used to select the most influential frameworks, those less frequently cited were not excluded if deemed interesting or innovative. We also added articles and books linked to more general concepts related to game-based learning or game design.

After selecting the relevant frameworks, we identified their main design elements.

Design elements were coded using the following steps:

1. Design elements explicitly presented in the selected frameworks were reproduced as is (verbatim).
2. Design elements appearing in texts or in graphics, but not explicitly presented, were added by creating a specific and relevant terminology. The terminology was proposed by one author (DJ) and confirmed by a second author (ML).
3. For frameworks dedicated to the design of serious games in broader fields than training, we assigned generic design elements to the corresponding specific element of the learning domain identified in steps 1 and 2 (eg, objectives were assigned to learning objectives). This was performed by one author (DJ) and confirmed by a second author (ML).
4. The design elements identified during steps 1 and 2 were then reviewed to regroup identical or duplicate items. That was done by one author (DJ) and confirmed by a second author (ML).

Results of steps 1 to 4 were then debated among all authors. Any disagreement was resolved by reaching consensus.

We grouped the identified design elements into categories according to their characteristics, the available literature regarding their use in serious game development, and the experience of the authors. The organization into categories was proposed by an author (DJ) and discussed among all authors and finally validated by the last author (ML).

We then converted design elements into *building blocks*, which represent the smallest units of the co.LAB framework. When similar or identical design elements were called differently, we decided upon a terminology that was then used to refer to the building blocks. For the sake of readability and consistency, some elements were renamed or merged. The proposition to rename or merge elements was issued by one author (DJ), discussed among all authors, and finally validated by the last author (ML).

Finally, once the co.LAB generic serious game design framework had been established, we determined the specifications required for its implementation in a collaborative web platform. This was achieved by converting the building blocks into *cards*: Each building block corresponds to a card with its collaborative functionalities. Each card can either be used or discarded according to its relevance for the design and development of a particular serious game.

During the entire process, we also took into account the authors’ experience in serious game development. Three of the authors (DJ, ML, and AH) belong to the AlbaSim research lab (Media Engineering Institute, University of Applied Sciences of Western Switzerland), which has been developing serious games for more than a decade. This lab has conducted serious game projects from design to implementation in many different educational fields such as emergency triage at hospital, cardiac clinical evaluation, oncology care, project management, computer education, energy management, or crime scene investigation [31-36]. Another author (LS) has conducted serious game projects in fields like resuscitation and COVID-19 infection prevention and control at the Geneva University Hospitals [37,38].

## Results

### Literature Analysis

The review and analysis of existing design frameworks confirmed that no single model or theory can currently be applied to the design of every kind of serious game. This is best explained by the fact that serious games may be of such different types and used in such different learning paradigms and contexts that a unique design framework may not be possible. This is confirmed by Plass et al [27] in their analysis of theoretical foundations of game-based learning: “It does not appear likely that a single theory will emerge that can guide the design of games for learning in general.” This is also in line with our experience in serious game development.

Another observation was that not all serious game design frameworks have the same objectives. Some are more oriented toward serious game design elements [6,19,25], some more toward the design and development process [39], some cover both design elements and design process [16], and others are more concerned with theoretical foundations of game-based learning [8,27]. While most frameworks are conceived to guide the design of serious games for learning purposes, some of them are intended for the development of serious games for generic purposes (including learning, but not only) [16,40]. Most frameworks focus on game design but do not take into consideration the learning scenario in which the game should be integrated.

A synthesis of the design elements identified in the selected frameworks can be found in Table 1. In this table, frameworks are presented in descending order of number of citations. This may be a sign of the influence of the framework, but not necessarily of its intrinsic quality.

Some elements are present in most frameworks, such as play, interactivity, and feedback. Some interesting elements are however only present in a few frameworks, such as usage context (ie, the context in which the game will be used, designing the simulation model, or defining learner specifications).

**Table 1:** Design elements extracted from existing serious game design frameworks and methodologies, which are presented in descending order of number of citations (from left to right).

Design elements	Plass (2015) [27]	Mitgutsch (2012) [40]	Yusoff (2009) [25]	Aleven (2010) [26]	de Freitas (2006) [28]	Rooney (2012) [8]	Marne (2012) [19]	Marfisi-Schottman (2012) [39]	Verschueren (2019) [16]	Vermeulen (2017) [41]
Learning objectives		x	x	x			x	x		x
Learning functions	x									
Learning foundations	x					x			x	
Learning activities			x					x		
Pedagogical scenario				x	x		x	x		
Learning mechanics	x									
Learning incentives	x									
Learning assessment	x							x	x	
Knowledge content	x	x	x						x	x
Game goals and rules				x					x	
Structure and progression			x				x	x		x
Game mechanics	x	x	x	x					x	
Decorum, sounds, aesthetics	x	x		x	x		x	x	x	
Narrative	x	x							x	
Game assessment	x							x		
Simulation model							x			
Fidelity					x	x				
Game incentives	x		x							
Usage context		x			x					x
Learners' specifications		x			x				x	
Play, interactivity, feedback	x	x	x	x	x	x	x	x	x	x
Immersion, motivation	x		x		x	x				

Besides the identification of these design elements, another key component was brought out through our analysis of the literature. Indeed, most frameworks emphasize the importance of developing an integrated system that includes and links gaming and learning aspects [6,8,19,25,26]. The successful alignment between game mechanics and learning mechanics is thus highlighted as an essential feature for the success of serious games [25,27,42,43].

Finally, many frameworks also tackle the development process, which is presented as both multidisciplinary and iterative [16,27,44]. But none of the selected frameworks provide specific project management functionalities.

### Specifications for a Generic Serious Game Design Framework

Since the aforementioned analysis confirmed that no currently available framework is truly exhaustive and as the diversity of serious game designs must be acknowledged, a single design method can hardly be developed. We therefore concluded that the generic framework we sought to create should be a methodology (ie, a set of methods and best practices at the disposal of a design and development team), rather than a method.

This generic framework should include elements common to most serious game designs but be *adaptive* to allow for specific designs. The framework should also foster collaboration between the various specialists involved in the design project. This could be achieved by allowing the design team to visualize the links between design elements throughout the game design process. This requires the development of a *systemic* framework.

#### Adaptive Features

Design specificities result from factors such as the type of serious game (which can include a simulation model, narratives, or neither of these features), usage context (ie, children or professionals, face-to-face, or online), and final objectives (eg, education, education and research, summative assessment).

Design specificities support the idea of developing an adaptive framework that would allow design teams to adapt the general model provided by the framework to their current project. This means that, depending on the design context and objectives, it should be possible to merge, add, or remove building blocks in the model.

The basic framework should therefore provide the main building blocks of most serious game designs (for example, pedagogical objectives, pedagogical scenario, or game design), but the design team should then be able to adapt these building blocks to their specific design project.

#### Systemic Features

Serious game designs include many elements, all of which are interconnected. A successful design depends as much on the quality of each element as on the relevance and adequacy of the links between these elements [8,19,25,26,42]. It is the relationships between the elements that give the final product its coherence. We thus hypothesized that the paradigm of complex systems and systemic modelling [45,46] would be a suitable approach for the design of serious games as complex systems.

This supports the idea of developing a framework that shall encourage a systemic approach. The systemic features should provide a vision of the serious game design as a whole made up of interacting elements. This implies that the framework should aim to provide both an overview of the building blocks used for the design of the serious game and of the relationships between them.

### The co.LAB Framework

In accordance with our previous findings, we defined the *co.LAB framework* as a methodological framework for serious game design.

In order to bring together in a structured vision all the design elements identified in Table 1, we defined 5 main categories: (1) Context and objectives, (2) Game design, (3) Learning design, (4) Mechanics, and (5) Assessment.

Each design element has been assigned to a category. For the sake of readability and consistency, we rearranged the design elements into 23 building blocks. Most design elements were reproduced verbatim. To be consistent with the category to which they were assigned, some elements were either renamed (“structure and progression” became “game structure”) or merged (fidelity and simulation model). Discussions between authors led to the addition of one building block (“game universe”) that was not clearly mentioned in any of the selected frameworks but found in general game design literature [47]. The proposed building blocks are not intended to represent all the potential elements that could be used for the design of any serious game. Rather, these building blocks represent the basic elements of standard serious game design. They are intended to be customizable to fit specific projects.

**Figure 1:**
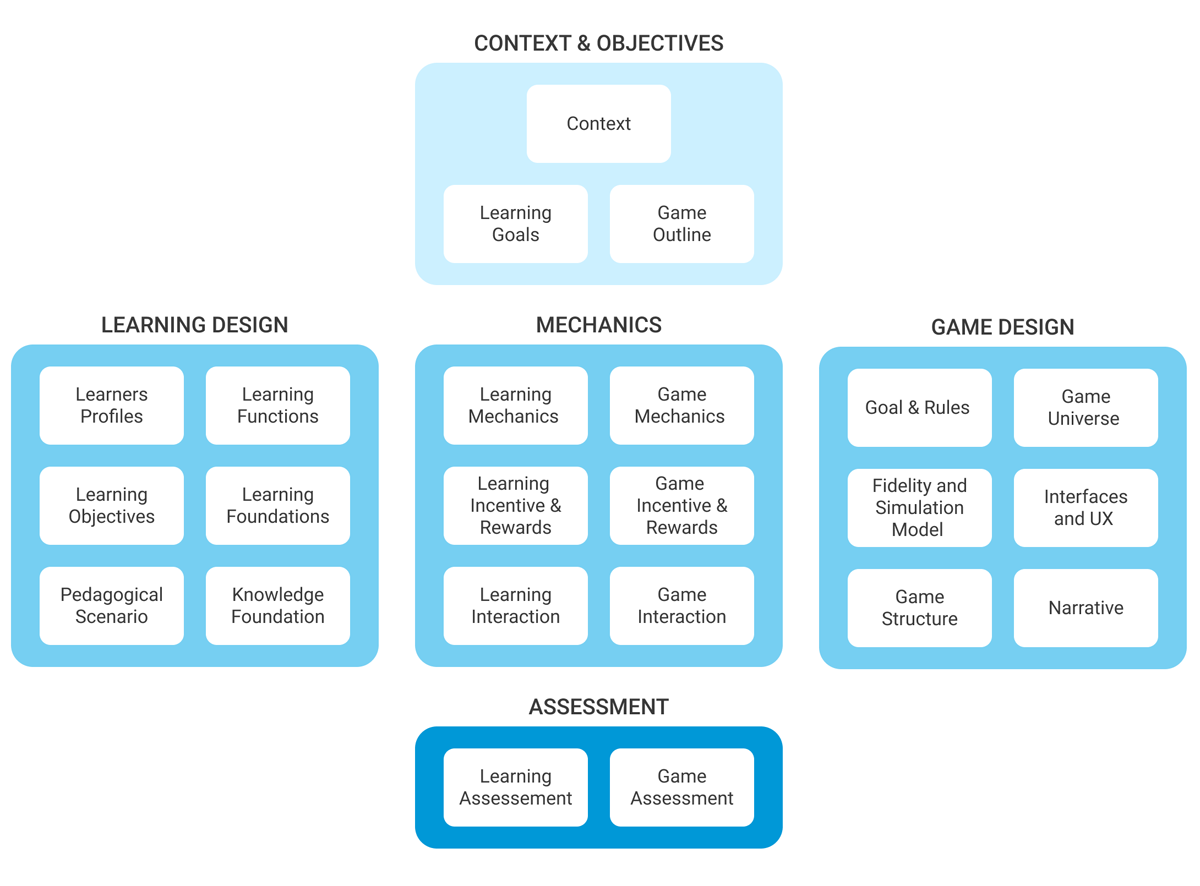
The co.LAB framework with its 5 categories and 23 basic building blocks. Building blocks can be added, adapted or removed according to the serious game design considered.

Figure 1 presents the identified building blocks grouped into categories, which will be described in the following section.

#### Context and Objectives

The “Context and objectives” category is intended to give an overview of the problem at hand and a first idea of the solution.

Context includes a description of the environment in which the serious game will be used: classrooms or other premises, available technology, number of participants, available class hours, and all other initial constraints that should be taken into account [40,48].

Learning goals are used to give a general definition of the knowledge and skills that participants should acquire by playing the serious game. As for the game outline, defining learning goals early in the development process helps give a direction to the project even though they may evolve. A single sentence summarizing these goals can also be used for external communication.

The game outline is a short description of the serious game. It often takes the form of “The player takes the role of a __ which is in a __ (context/situation/environment). His/her objectives are __. For this, he/she must __.” The game outline can evolve during the course of the development, but its definition from the start of the project gives a direction to the development team. The game outline will often be used for communication with people outside of the project (such as stakeholders).

#### Learning Design

Learning design aims at defining and designing the learning aspects of the serious game.

Defining the profiles of the participants, including their digital literacy [49,50], interest in learning the subject matter, and gaming and simulation experience will help adapt the content of the game and its mode of delivery [16,48].

Regarding learning functions, by definition, serious games are designed for a primary purpose other than pure entertainment [51]. When designed for learning purposes, a clear definition of the learning functions is necessary to achieve the intended goal. Development teams must define whether the serious game will be used as an exercise designed to test or apply existing knowledge or skills, to support knowledge or skill acquisition, or to prepare for future courses [27].

Learning objectives (or learning outcomes) are the results of breaking down learning goals into measurable sub-elements. They define what participants should have learned by the end of the serious game training sessions and are often stated as “at the end of the serious game, participants will be able to...” The learning objectives are the basis for building the serious game structure and content, defining appropriate teaching and learning methods, and designing learning assessment modalities. They can be used to inform students of what they are expected to learn.

For the learning foundations, appropriate learning theories and pedagogical modalities must be chosen. Depending on learning objectives, an appropriate learning theory could be behaviorist, constructivist, or socio-constructivist [8,27]. They can be declined in several pedagogical approaches such as experiential learning or problem-based learning [8]. The choice of appropriate learning theories and pedagogical modalities is a necessary condition for the achievement of learning objectives. For example, if the main learning objective is to develop practical palpation skills for clinical assessment and the pedagogical modalities are “observation,” there will be an inconsistency that may prevent the learner from acquiring the intended skill.

For the knowledge foundations, the objective is to identify and validate the content related to the knowledge and skills participants are expected to acquire. This is the field of professional expertise. For example, evidence-based triage rules and processes should be the knowledge foundations of a serious game designed to teach emergency triage procedures. The identification of relevant knowledge foundations should be performed through a review of the relevant literature or of professional standards in collaboration with subject matter experts. It will also be necessary to define how the serious game will enable knowledge or skill acquisition [16,27].

The serious game should not be a stand-alone intervention but rather be embedded within a pedagogical scenario. The pedagogical scenario is therefore related to the general structure of the course or of the study program. The pedagogical scenario can be made up of a sequence containing game sessions, theoretical lectures, and personal working time. Depending on the pedagogical scenario, a subscenario can be required to support the use of the serious game. This subscenario generally includes 3 phases: prebriefing, orchestration of the game, and debriefing [52-55]. The activities taking place around the game (prebriefing and debriefing) are as important as the game itself.

#### Mechanics

Mechanics are at the core of the framework. They form the link between learning design and game design. In line with the model by Arnab et al [42], learning objectives should be linked to learning mechanics, which should be linked to game mechanics. Game mechanics should then be linked to game goals, rules, and structure.

The development team must decide upon the main learning mechanics that will be implemented in the serious game. We agree with the definition given by Plass et al [56], who defines learning mechanics as “patterns of behavior or building blocks of learner interactivity, which may be a single action or a set of interrelated actions that form the essential learning activity that is repeated throughout a game.” Learning mechanics can include activities such as remembering, understanding, applying, analyzing, evaluating, or creating. Learning effectiveness increases when learning and game mechanics are aligned with learning objectives [42,56-58]. This leads participants to develop and exercise their cognitive abilities throughout the game to reach its ultimate goal.

Game mechanics are the set of actions repeated by the player throughout the game [59] and are therefore the basic elements of interactivity. A game can include a single game mechanic (such as only shooting, jumping, or answering questions) or an integrated set of game mechanics (for example, moving around freely while answering questions and collecting objects). In serious games, game mechanics have a double objective, resulting in 2 constraints: (1) engaging participants in taking part in the game and (2) ensuring consistency with learning mechanics. An incorrect choice of game mechanics can therefore quickly lead to failure in serious game implementation.

Learning and game incentives and rewards are used to support participant engagement and motivation. Incentives can be either *intrinsic* or *extrinsic* [58,60]. Intrinsic incentives are linked to game play and learning outcomes, whereas extrinsic incentives are not directly related to these elements. The most commonly used extrinsic incentives are points, badges, and trophies. Intrinsic incentives are more effective than extrinsic ones in achieving the learning objectives or any other intended goal. Indeed, it has been pointed out that gamification mechanisms purely based on rewards and on extrinsic motivators only bring short-term benefits and can be worthless or even harmful in the long run [58]. Intrinsic incentives may be harder to implement but are more beneficial. They can come from 3 sources: (1) mastery (learning to the point of feeling mastership regarding a specific knowledge or skill), (2) autonomy (being able to choose between several paths), and (3) relatedness (not feeling alone, feeling connected to others or to the situation) [61].

It is through interaction with the mechanics of play and learning that participants advance in the game and acquire knowledge and skills. To be successful, the interactions designed by the development team must result in both meaningful play and meaningful learning. Salen et al [59] defined meaningful play as emerging from players’ actions that are discernible (players receive feedback) and integrated into the game play (players understand how their actions influence the course of the game).

Meaningful learning, as opposed to rote learning, is achieved when the learner is actively engaged in the learning process and the newly learned information is connected with previous knowledge. Mayer [62] argued that meaningful learning occurs when learners build knowledge for successful problem solving. In serious games, meaningful learning may be achieved when participants need to acquire new knowledge for solving problems encountered in the game. Meaningful learning may happen either inside the game or outside the game, for example during the debriefing phase.

The successful implementation of meaningful play and meaningful learning leads to what could be called *meaningful serious gaming*.

#### Game Design

The game design includes the detailed description of all the elements that form the serious game.

Regarding goals and rules, setting a goal is essential for developing the pleasure and motivation to play. The goal of the game should be understandable, concrete, simple, clear, achievable, and rewarding if achieved [47,59]. A game is an artificial conflict to be resolved by the player [59]: The development team will have to decide which activities and interactions the player will be allowed to perform (game rules) to achieve the objective (game goal).

The game universe corresponds to the world in which the game will be played. It may be a fictional world or a simulation of the real world. The game universe should be consistent with the learners' profiles.

Regarding the fidelity and simulation model, a simulation is a simplified representation of reality that seeks to achieve fidelity. Different kinds of fidelities have been described, all of which are used to enhance realism: sensory fidelity (audio-visual), narrative fidelity (dialogues, story), and cognitive fidelity (reflections that players make in the serious game) [8,63]. The types of fidelity chosen by the development team must be consistent with pedagogical objectives.

User interfaces (UI) and user experience (UX) are related to what the player will see and experience. They will impact the emotional feeling of the game and the pleasure of playing [26,27]. The graphical design and sounds must be aligned with the game universe and the desired fidelity. UX and game usability must be considered according to the context of usage and learners’ profiles.

The game structure should include the description of both the game and the learning progression. If there are prerequisite relationships between knowledge chunks acquired during the game, they will have to be taken into account when defining the game-learning sequences [19,41]. A progression that is too difficult or too slow will decrease the player’s motivation. This is in line with the concept of flow described by Csikszentmihalyi [64]. The progression must be thought of as much from a game perspective by game designers as from a learning perspective by educators [19].

Narratives are the content and structure of the story. They can include information given by a narrator, dialogues between the player and characters, and emails. Writing dialogues and narratives means creating an interactive scenario that will evolve according to the player's choices. The quality of narratives will depend on the number and quality of choices and on the number and quality of feedback.

#### Assessment

How the game and its objectives will be evaluated is part of the overall design. This may include game assessment by participants, learning assessment within the game itself, or assessments outside the game. If a research project is considered, the research questions should be clearly defined, and research protocols should be established and registered. This will help determine which data and indicators will ultimately be needed and how data processing and visualization should be carried out. Care must also be taken to ensure compliance with personal data protection regulation. Consent mechanisms and the need for ethics approval must also be considered.

### Visual Organization of the Categories

**Figure 2:**
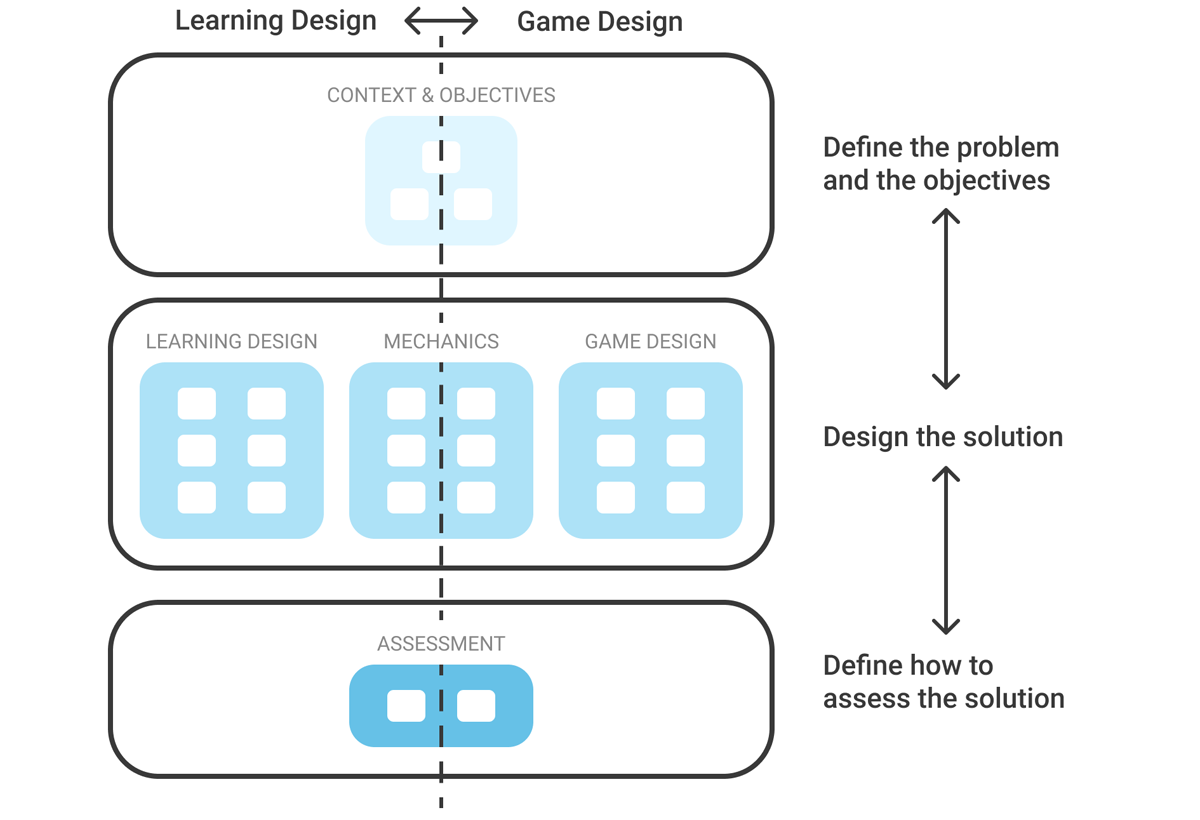
Structured vision of the 5 serious game design categories.

By grouping design building blocks into 5 categories, the framework aims at providing a structured view of the game design. This view enables all members of the development team to focus on the building blocks on which they are working (pedagogical engineers may focus on learning design, while graphic designers on user interfaces) while simultaneously providing an overview of the project and of the relationships between the building blocks. The categories are structured both vertically and horizontally (Figure 2).

#### The Game and Learning Vision

Traditionally, serious game design is viewed as a blend of learning and game design. In Figure 2, the left side of the framework corresponds to the learning design, and the right side corresponds to the game design. As in most design models [44,56], this vision emphasizes the inclusion of game and learning designs in serious game design. The Mechanics category can be seen as linking them together.

#### The Project Vision

The co.LAB framework can also be viewed from top to bottom. The upper section defines the problem and the overall objectives of the project. The middle section defines the solution: the game and the associated learning concept. The lower section defines how the solution will be evaluated both from the game and learning perspectives. This may in some cases be similar to the “success criteria” defined in project management theories.

## Discussion

### Implementation in a Collaborative Web Platform

To support the collaborative work of a multidisciplinary development team often geographically dispersed and to enable all partners to have a common and up-to-date vision of the design, the co.LAB framework should be implemented in a collaborative web platform. In this section, we discuss the specifications and requirements for this implementation.

**Figure 3:**
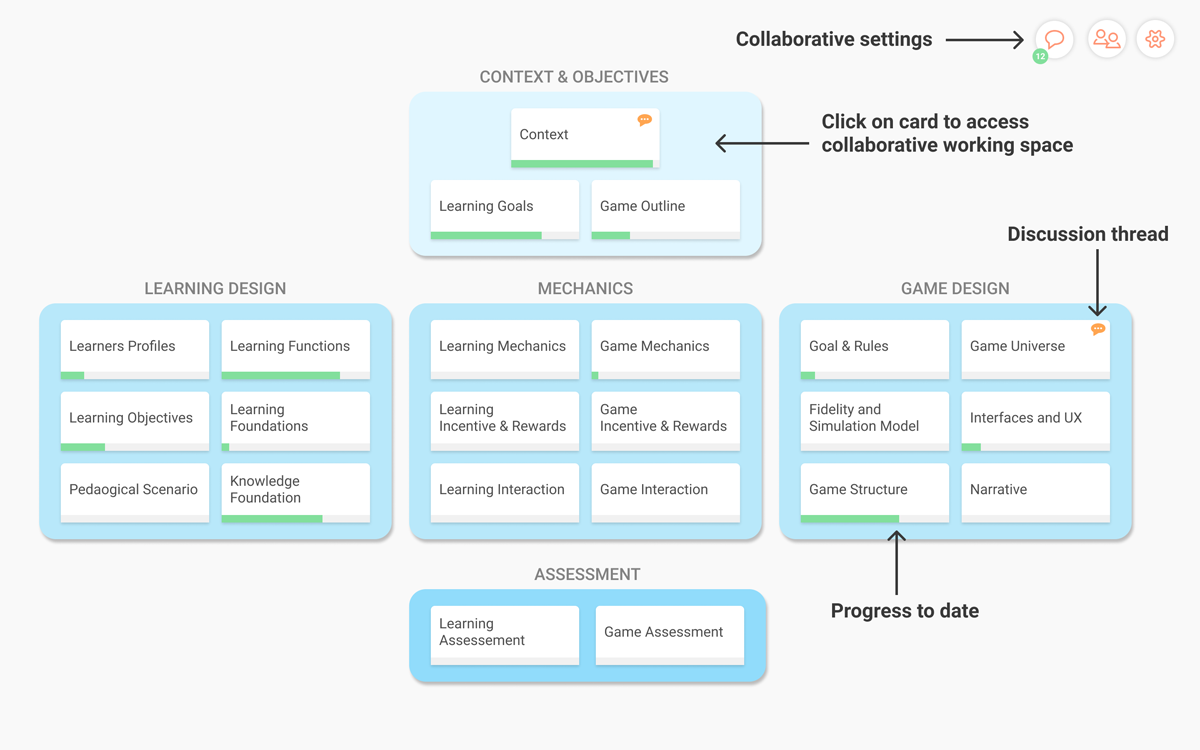
Wireframe for the implementation of the co.LAB framework into a web platform: main project page.

The web platform should be open source and open access, thus allowing any serious game development team to use it freely. At the beginning of a serious game project, the development team should access a visual initial design template based on the co.LAB framework. Figure 3 shows how the main screen of a design project based on the co.LAB framework could look. As team management or project monitoring functionalities would be useful to help allocate resources and quickly check on the project’s advancement, they should be embedded in the web platform.

#### Cards as a Collaborative Workspace for Each Building Block

**Figure 4:**
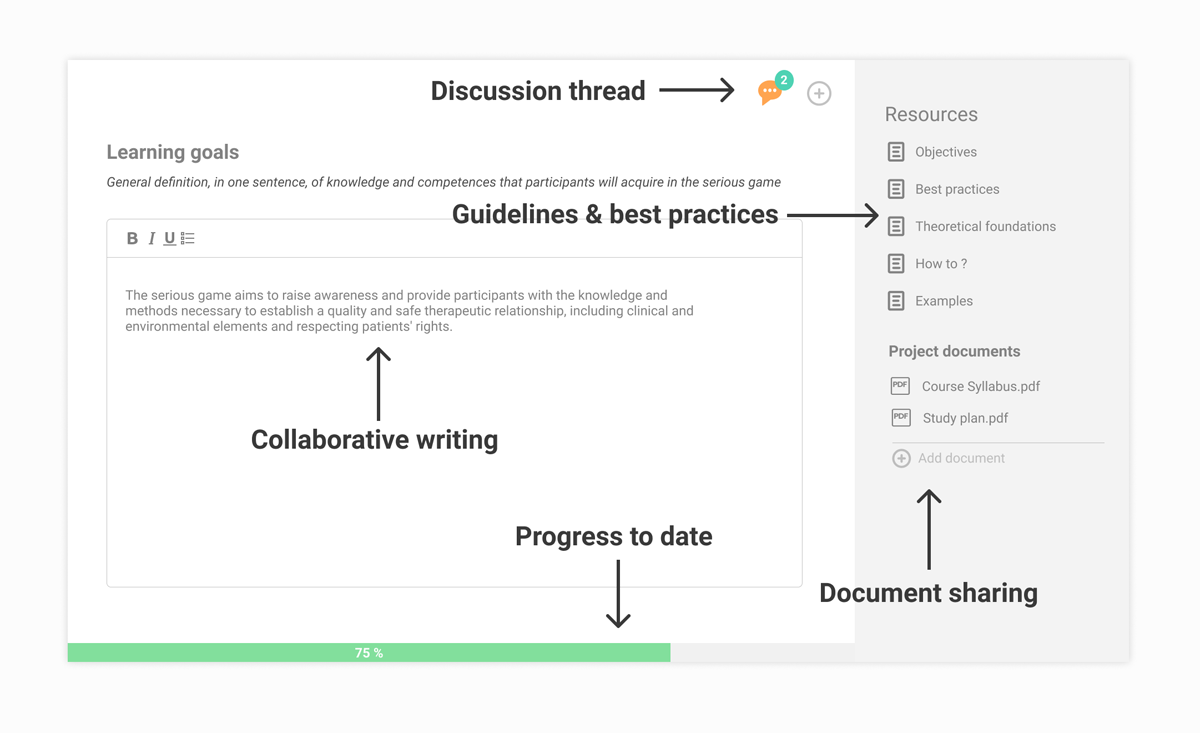
Wireframe of a Card, with collaborative working spaces and access to resources.

In the web platform, each building block (eg, context, game outline) should be implemented as a *Card*. By clicking on the card, the development team would access a dedicated collaborative workspace. The workspace of each card should provide teamwork functionalities such as collaborative writings, discussion threads, modification proposals, and document sharing. Each card should be complemented with methodological resources such as definitions, best practices, tools, or theoretical references (Figure 4).

#### Implementation of the Adaptive Features

At the beginning of a serious game development project, the development team would be provided with a basic template including the most frequently used cards. This basic structure covers the conventional elements of standard serious game design and is suitable for use in this form by junior development teams. More experienced development teams could adapt this basic model by adding, modifying, or deleting cards. Cards could either be added from a store of already available optional cards or created from scratch by the development team.

Providing abilities to select the most relevant cards, to discard others, and to create missing cards should allow the initial template to be adapted toward an already existing model or to customize it for a specific serious game design.

The framework’s basic template should also be adaptive. Based on the analysis of the traces of use of different development teams, the platform administrators should be able to make the model evolve.

#### Implementation of the Systemic Features

**Figure 5:**
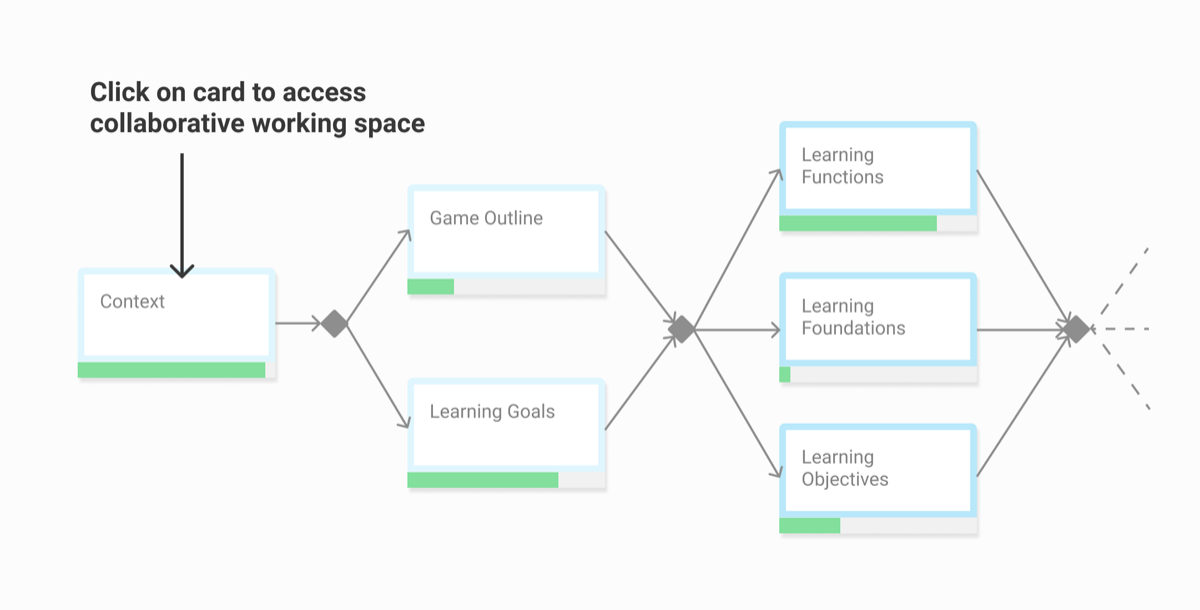
Activity network diagram view, emphasizing precedence dependency relationships.

**Figure 6:**
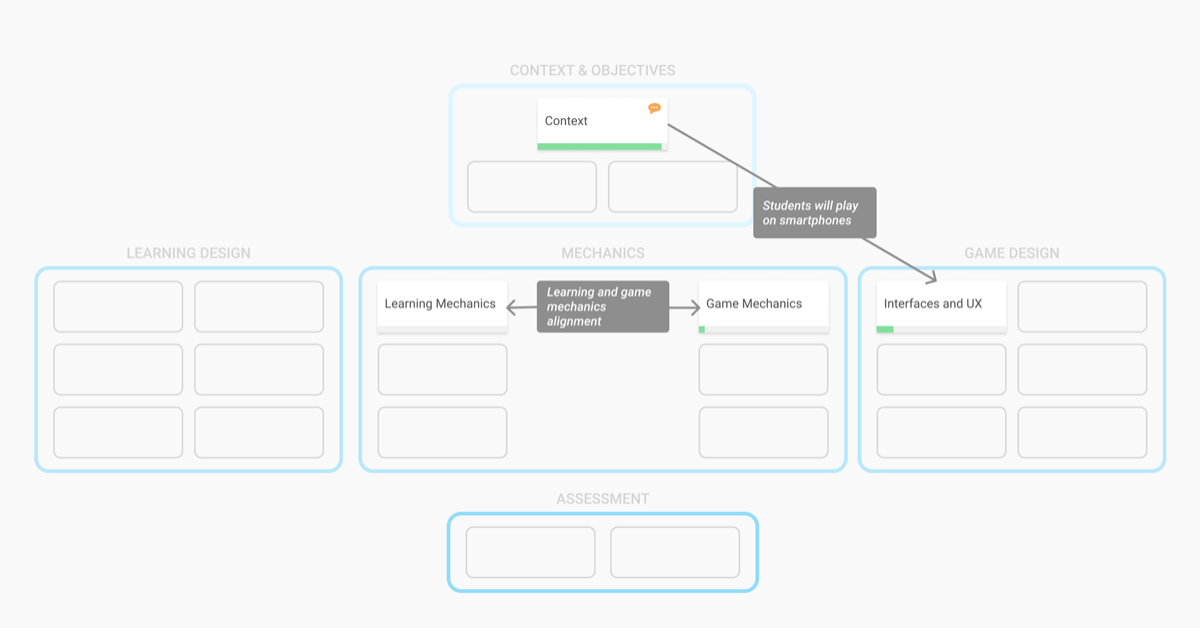
Causality and interdependence relationships.

Figure 3 shows a default view of the model in which cards are grouped by categories. The platform should also allow the development team to develop a systemic view of the project by creating links between cards. Specific views should then be generated to allow team members to visualize such links (Figures 5 and 6).

There are 3 main types of relationships between cards. The first, precedence dependency relationship, is used to create the activity network diagram and for scheduling. The second, causality, is used to inform that something defined in 1 card must be taken into account in another. The third, which may be called interrelationship, indicates that 2 cards should be seen as a coherent whole. This interrelationship can also be described as a bidirectional causality.

Each relationship could also have its own collaborative workspace, with best practices and resources. Some relationships could already be defined in the initial model. For instance, as an interrelationship is mandatory to link game mechanics and learning mechanics, this relationship could already be available from the start of the project. The collaborative workspace attached to this relationship would provide best practices guidelines related to the alignment of game and learning mechanics.

The development team should also be able to create specific relationships, as, for example, a link between the context and user interfaces with a remark that the game should be playable on smartphones.

#### Supporting all the Project Phases

**Figure 7:**
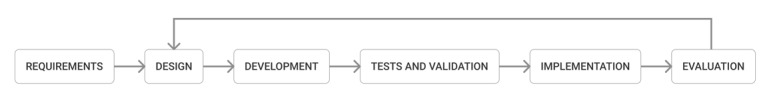
General view of the iterative development process.

The web platform should support the development team all along the course of the project, from design to development and evaluation. In Figure 7, we propose a serious game development process that should be implemented in the web platform. This process is a quite classical adaptation of traditional agile project management. Using this kind of agile approach for serious game development is endorsed by Verschueren et al [16] and Alvarez et al [44] and by the authors’ experience.

**Figure 8:**
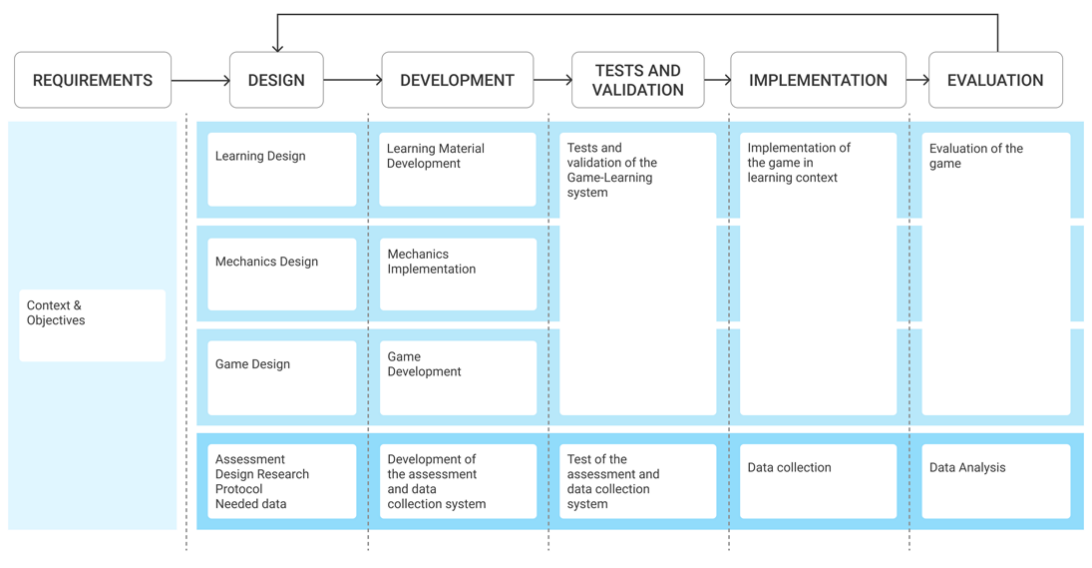
Extension of the co.LAB framework along the entire serious game life cycle.

The co.LAB framework presented in this paper focuses on the Requirements and Design phases of the process. It is however possible to extend the framework to encompass the whole process (Figure 8).

By providing a coherent link between building blocks during all phases of the project, the co.LAB framework could be used throughout the life cycle of the serious game.

The different visualizations of the building blocks (grouped into categories, activities diagram, relationships, project life cycle) correspond to different ways of approaching the same problem of serious game design and development. These various visualizations should allow for a shared understanding and a consistent approach throughout the design and development process.

### Principal Findings

The co.LAB serious game design framework was created by identifying design elements commonly used to design and develop serious games. These design elements were defined and synthesized to create design building blocks, which were grouped in 5 categories: (1) context and objectives, (2) game design, (3) mechanics, (4) learning design, and (5) assessment. The framework recognizes the diversity of serious game design and is conceived to be adapted to specific contexts, by adding or removing building blocks.

The different visualizations of the building blocks (grouped into categories, activity network diagram, causality relationships, project life cycle) correspond to different ways of approaching the problem of serious game design and development. These various visualizations should allow for a shared understanding and a consistent approach throughout the design and development process.

The co.LAB framework is designed to be implemented in a collaborative web platform, with implementation recommendations that should support teamwork and knowledge sharing within a multidisciplinary team and favor an adaptive and systemic approach. The co.LAB framework may be used as a guideline along all project phases, from requirements to design, development, tests, implementation, and evaluation.

### Future Work

The co.LAB framework is currently being implemented in a collaborative web platform.

Guidelines related to each card are currently being developed and will gradually be implemented in the platform. Their development is based on a literature review and on the authors’ experience. Their relevance will be evaluated by end users, and they will be updated according to the feedback obtained.

We plan on testing this framework and the web platform on which it is being implemented through the development of different kinds of serious games. This should allow us to troubleshoot the platform and identify the most important areas of improvement.

In a future version, we plan on implementing electronic assistance to help users find the most suitable combination of cards depending on their specific serious game project.

### Limitations

The co.LAB framework is not based on a complete systematic review of serious game design frameworks, but rather on a review of the most influential and most relevant frameworks according to the authors’ opinions and experience. However, should any particular design element be missing from the current version of the framework, its adaptive features should allow development teams to include them.

The co.LAB framework is also based on the authors’ experience in serious game design and development. Even though the authors have developed many serious games in different subject matters and contexts, their experience is still limited and does not include all kinds of serious game development. Once again, the adaptive features of the co.LAB framework should mitigate this limitation

Finally, the co.LAB framework has been neither fully implemented on a web platform nor used to create a full-fledged serious game yet. Even though its development was theory-driven and based on relevant and authoritative references, it should be thoroughly tested before its routine use can be recommended.

### Comparison With Prior Work

In comparison with previous work, the co.LAB framework proposes some novelties. First, this framework recognizes the diversity of serious game design and is intended to be adaptable to specific contexts. Second, the framework was designed to be implementable in a collaborative web platform. Finally, this implementation in the web platform is based on a systemic approach of the design process.

### Conclusion

By offering a structured view of fundamental design elements, the co.LAB framework should facilitate the design and development of serious games through a collaborative, multidisciplinary, adaptive, and systemic approach. The ability to visualize the building blocks and their relationships from different standpoints should allow for a shared understanding and a consistent approach throughout the design and development process. The co.LAB framework was designed to be implemented in a collaborative web platform that is currently under construction. Once fully implemented, the actual usability and effectiveness of this new framework should be thoroughly tested.

## Abbreviations

UI: user interface
UX: user experience
